# Relationship between plasma YKL-40 levels and endothelial dysfunction in chronic kidney disease

**DOI:** 10.3906/sag-1804-169

**Published:** 2019-02-11

**Authors:** Gül Sema KESKİN, Özant HELVACI, Çağrı YAYLA, Özge T. PAŞAOĞLU, Çağlar KESKİN, Turgay ARINSOY, Ülver B. DERİCİ

**Affiliations:** 1 Department of Oncology, Faculty of Medicine, Başkent Unıversity, Ankara Turkey; 2 Department of Nephrology, Faculty of Medicine, Gazi University, Ankara Turkey; 3 Department of Cardiology, Yüksek İhtisas Hospital, Ankara Turkey; 4 Department of Biochemistry, Faculty of Science, Gazi University, Ankara Turkey; 5 Department of Endocrinology, Faculty of Medicine, Ankara University, Ankara Turkey

**Keywords:** YKL-40, CKD, hemodialysis, atherosclerosis, endothelial dysfunction

## Abstract

**Background/aim:**

We aimed to evaluate the relationship between YKL-40 and endothelial dysfunction in chronic kidney disease.

**Materials and methods:**

Twenty-nine hemodialysis patients, 101 patients with nondialytic (stage 2, 3, 4, and 5 ND) chronic kidney disease (CKD), and 38 healthy individuals as a control group were included. YKL-40 levels were measured by ELISA. Endothelial dysfunction was indirectly measured by flow-mediated dilatation percentage (FMD) in the brachial artery.

**Results:**

YKL-40 levels were higher in CKD patients than controls and highest in HD patients (P = 0.001). FMD values were lower in nondialytic CKD patients and lowest in HD patients (P = 0.001). YKL-40 negatively correlated with eGFR and FMD values (r = –0.674 and r = –0.471, respectively).

**Conclusion:**

This study shows that YKL-40 increases with CKD stage and is negatively correlated with FMD measurements.

## 1. Introduction

Chronic kidney disease (CKD) is an epidemic that affects 10%–15% of the modern world [1]. Numerous observational studies have demonstrated increased cardiovascular risk in the setting of CKD. Nearly half of the patients requiring renal replacement treatment die from cardiovascular diseases. Furthermore, age-adjusted cardiovascular mortality is 15–30 times higher in CKD patients than healthy subjects [1,2]. Traditional cardiovascular risk factors cannot clarify this significant difference. Therefore, nontraditional risk factors such as endothelial dysfunction, oxidative stress, and insulin resistance are being investigated. Endothelial dysfunction (ED) is related to cardiovascular disease and progression of CKD and it can be a causative factor for accelerated atherosclerosis in CKD patients [3,4]. ED was previously described as inadequate vasodilatation to certain stimuli. However, nowadays, we use a broader definition that includes proinflammatory and prothrombotic features of ED as well [5]. The degree of ED can be measured directly or indirectly. 

YKL-40 is a novel biomarker for acute and chronic inflammation. It is also known as human cartilage glycoprotein 39 or chitinase-3-like protein 1 (CHI3L1). It is released from vascular smooth muscle cells, synovial cells, articular chondrocytes, activated neutrophils, cancer cells, and macrophages. A major source of circulating YKL-40 is mature macrophages. The primary functions of YKL-40 remain mostly unknown, but it is currently thought that YKL-40 may have a role in the proliferation of chondrocytes and fibroblasts, differentiation of macrophages, migration and reorganization of vascular endothelial cells, remodeling of the extracellular matrix, and inflammation [6–8]. Activated macrophages obtained from early atherosclerotic lesions have been shown to express very high levels of YKL-40 [9]. High serum levels of YKL-40 are associated with cardiovascular disease and mortality [10,11]. Serum YKL-40 levels are also increased in patients with diabetes mellitus and hypertension [12]. Another study from our department revealed peritoneal dialysis and hemodialysis (HD) patients had elevated levels of YKL-40 compared to healthy subjects [13]. 

In this cross-sectional study, we aimed to demonstrate the relationship between YKL-40 and ED in patients with CKD(2–5) (nondialytic CKD, stages 2–5) and CKD5D (hemodialysis) patients.

## 2. Materials and methods

### 2.1. Patients

**Figure 1:**
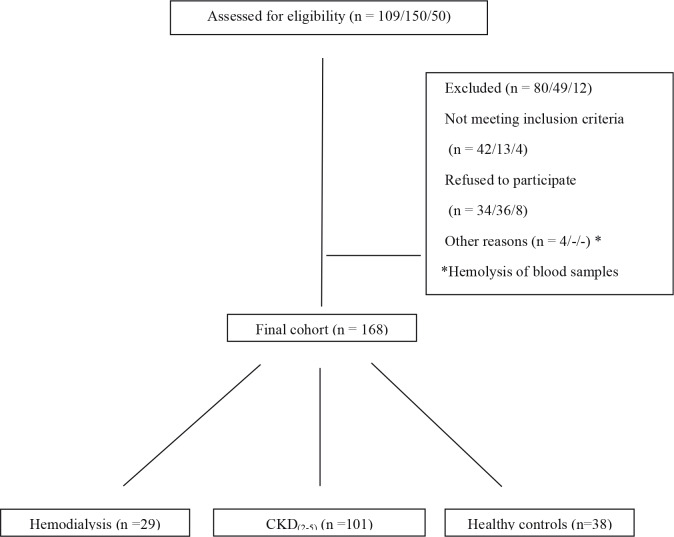
Patient selection (hemodialysis/CKD(2–5) patients/healthy controls respectively).

A cohort of 29 hemodialysis patients, 101 patients with nondialytic CKD (stages 2, 3, 4, and 5), and 38 healthy subjects were recruited between September 2012 and April 2013. All HD patients had a dialysis vintage of at least 3 months and all of them were on a three times weekly hemodialysis program for 4 h with standard bicarbonate dialysate and with synthetic membranes. CKD staging was done using the MDRD formula. The term “CKD(2–5) patient” was used to define patients with stage 2, 3, 4, and 5 (nondialysis) chronic kidney disease. For the control group, healthy subjects with no known diabetes, hypertension (excluded by two office measurements), cardiovascular disease, rheumatologic disease, or proteinuria (>200 mg/day) were selected. The local ethics committee of Gazi University, by the Declaration of Helsinki, approved the study (No: B.30.2.GUN.0.20/5847 - 14/9/2012); all participants gave their written informed consent before inclusion. Patient selection is presented in Figure 1.

Exclusion criteria for hemodialysis and CKD patients were as follows: 

a) Age <18 or age >75

b) Active infection

c) Pregnancy

d) Malignancy

e) Active or prior history of SLE, rheumatoid arthritis

f) Coronary intervention (angiography, ballooning, stenting, coronary artery bypass graft) in the last 6 months

Demographic and clinical data such as diabetes, hypertension, hyperlipidemia, cardiovascular disease, and smoking status were recorded. Antihypertensives were recorded as alpha blockers, beta blockers, diuretics, ACE in/ARBs, and calcium channel blockers. Statins, ezetimibe, and fibrates were all recorded as lipid-lowering treatments. All patients had a routine physical examination performed by the same physician. Body mass index (BMI) was calculated as weight in kilograms divided by height per square meter.

### 2.2. Biochemical measurements

Routine blood count, fasting glucose, BUN, creatinine, uric acid, albumin, high-density lipoprotein (HDL) cholesterol, low-density lipoprotein (LDL) cholesterol, triglyceride, total cholesterol, C-reactive protein (CRP), and YKL-40 levels were measured in the department of biochemistry and hematology with automated procedures. eGFR was calculated by the MDRD formula. All blood samples were centrifuged immediately at 3000 rpm for 5 min and stored at –20 °C until YKL-40 and CRP analysis. For YKL-40 levels, a commercially available ELISA kit (Cusabio Biotech., Wuhan, China) was used. Results are given as ng/mL. CRP was measured by the nephelometric method. All samples were obtained after at least 8 h of fasting. HD patients’ blood samples were collected before dialysis. 

### 2.3. Assessment of endothelial function by flow-mediated dilatation 

Flow-mediated dilatation (FMD) measurements were done on the dominant arm of healthy subjects and patients without arteriovenous fistulae. For the rest, measurements were done on the arm without the fistula. All measurements were done by the same physician, in the same place, and at room temperature. VIVID 7 (Vingmed-General Electric, Horten, Norway) ultrasonography was used for brachial artery sonography. At least 6 h of fasting, at least 12 h of abstinence from alcohol and caffeine, and 2 h of abstinence from nicotine was mandatory before measurements. End-diastolic brachial artery diameter (EBAD) was measured with the help of continuous electrocardiography monitoring. The mean of three consecutive measurements was accepted as the EBAD. At the same time, flow velocity was recorded. After this, the cuff was inflated to 50 mmHg higher than patients’ systolic blood pressure and was held for 5 min before being suddenly deflated. EBAD and flow velocity were rerecorded after 45 s (hyperemic response). FMD in response to reactive hyperemia was calculated as follows: 

FMD (%) = [after hyperemia EBAD – basal EBAD] / basal EBAD × 100 

### 2.4. Statistical analysis

Continuous variables were expressed as mean and standard deviations (SD) and categorical variables as frequency and percentage. Normal distribution patterns were tested with the Kolmogorov–Smirnov test. Independent samples t-test, the Mann–Whitney U test, and one-way analysis of variance (ANOVA) were used to test differences between groups when appropriate. Categorical variables were compared with the r2 test. Correlation analyses between continuous variables were performed using univariate Spearman correlation analysis. P < 0.05 was considered statistically significant. All statistical analyses were performed using SPSS 20.0 (IBM Corp., Armonk, NY, USA).

## 3. Results

Groups were similar regarding age, sex, and smoking status (P > 0.05). Diabetes, hypertension, cardiovascular disease prevalence, and smoker frequency were identical between nondialytic CKD and HD patients (P > 0.05). Hyperlipidemia, BMI, hypertension, and mean blood pressure were different between each group. The difference was statistically significant. Demographic features of the cohort are given in Table 1. 

**Table 1:** Demographic properties of study groups.

Variables	Control	CKD	HD	P-value
Age (mean ± SD)	49.39 ± 11.7	53.10 ± 11.50	48.14 ± 14.52	0.053
Sex (female)	19 (50%)	47 (46.5%)	10 (34.5%)	0.413
BMI (kg/m2)	26.69 ± 3.67	28.90 ± 4.75	24.57 ± 4.50	<0.001
CVD (present)	-	18 (17.8%)	3 (10.3%)	0.406
Diabetes (present)	-	42 (41.6%)	7 (24.1%)	0.136
Hypertension (present)	-	88 (87.1%)	21 (72.4%)	0.083
Hyperlipidemia (present)	14 (38.9%)	73 (72.3%)	18 (62.1%)	0.002
Smoking (active smoker)	4 (10.5%)	16 (16.2%)	8 (27.6%)	0.174

CVD: Cardiovascular disease, BP: blood pressure, HDL: high-density lipoprotein, CRP: C-reactive protein, FMD: flowmediated dilatation, CKD: chronic kidney disease (stages 2–5, nondialysis), HD: hemodialysis.

Hemoglobin, albumin, HDL, and FMD (%) were significantly lower in nondialytic CKD and HD patients (combined) compared to controls. YKL-40, fasting glucose, and uric acid levels were significantly higher in HD and nondialytic CKD patients than controls. Nondialytic CKD and HD patients had higher blood pressure, triglycerides, total cholesterol, and CRP levels compared to controls. LDL values were not different between groups. Results are shown in Table 2. 

**Table 2:** Comparison of study parameters (control group vs. CKD+HD).

Variables	Control	CKD+HD	P-value
BP (mean ± SD) Systolic Diastolic Mean	118.94 ± 10.6 72.76 ± 7.50 88.15 ± 7.67	129.68 ± 19.50 77.64 ± 12.56 94.99 ± 13.95	0.000 0.024 0.002
Hemoglobin (g/dL)	14.00 ± 1.24	12.92 ± 2.06	0.002
Albumin (g/dL)	4.30 ± 0.22	4.1 ± 0.48	0.001
HDL (mg/dL)	48.54 ± 9.42	42.1 ± 11.05	0.001
CRP (mg/L)	3.18 ± 2.39	9.07 ± 16.00	0.026
Glucose (mg/dL)	90 ± 9	115 ± 51	0.000
Uric acid (mg/dL)	5.13 ± 1.23	6.55 ± 1.64	0.000
FMD (%)	13.24 ± 6.34	4.46 ± 3.65	0.000
YKL-40 (ng/mL)	31.73 ± 21.12	61.68 ± 24.72	0.000

BP: Blood pressure, HDL: high-density lipoprotein, CRP: C-reactive protein, FMD: flowmediated dilatation, CKD: chronic kidney disease (stages 2–5, nondialysis), HD: hemodialysis.

When HD and nondialytic CKD patients were compared, nondialytic CKD and HD patients had similar systolic blood pressures, fasting glucose, uric acid, and CRP levels. However, nondialytic CKD patients had elevated diastolic and mean blood pressures compared to HD patients. Hemoglobin, albumin, HDL, LDL, total cholesterol, and FMD (%) were significantly lower in HD patients than nondialytic CKD patients. No difference between triglycerides was observed. YKL-40 levels were significantly higher in HD patients. Results are shown in Table 3. 

**Table 3:** Comparison of study parameters (CKD vs. HD).Comparison of study parameters (CKD vs. HD).

Variables	CKD	HD	P-value
BP (mean ± SD) - Systolic - Diastolic - Mean	131.11 ± 17.97 79.38 ± 11.94 96.62 ± 13.04	124.14 ± 24.13 70.86 ± 12.82 88.62 ± 15.72	0.086 0.001 0.005
Hemoglobin (g/dL)	13.40 ± 1.94	11.05 ± 1.36	.000
Albumin (g/dL)	4.17 ± 0.51	3.83 ± 0.42	0.041
HDL (mg/dL)	42.89 ± 11.10	36.29 ± 9.26	0.003
CRP (mg/L)	7.50 ± 9.54	14.66 ± 28.78	0.33
Glucose (mg/dL)	118 ± 54	103 ± 37	0.08
Uric acid (mg/dL)	6.64 ± 1.75	6.17 ± 1.04	0.067
Proteinuria (mg/day)	1598 ± 2458	N/A	N/A
FMD (%)	4.85 ± 3.76	2.95 ± 2.78	0.014
YKL-40 (ng/mL)	55.76 ± 23.09	83.92 ± 16.88	0.000

BP: Blood pressure, HDL: high-density lipoprotein, CRP: C-reactive protein, FMD: flowmediated dilatation, CKD: chronic kidney disease (stages 2–5, nondialysis), HD: hemodialysis, N/A: not available.

### 3.1. Correlation analysis

**Figure 2:**
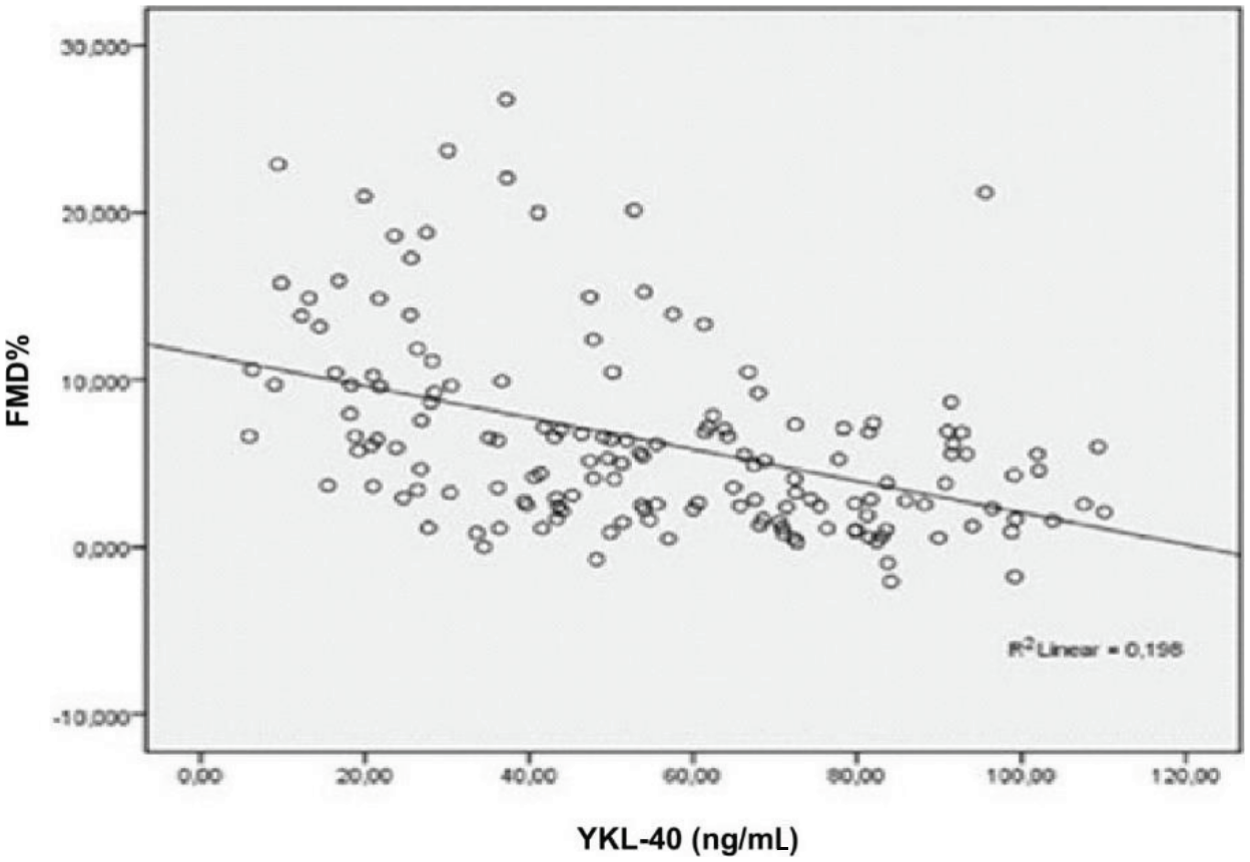
Correlation between FMD% and YKL-40 (P < 0.001, r: –0.471).

**Figure 3:**
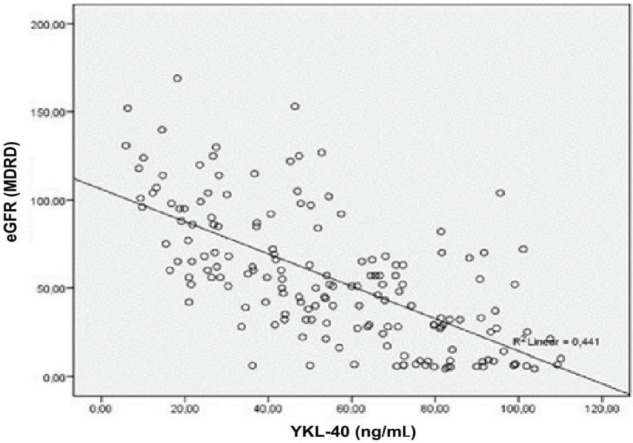
Correlation between eGFR and YKL-40 (P < 0.001, r: –0.674).

**Figure 4:**
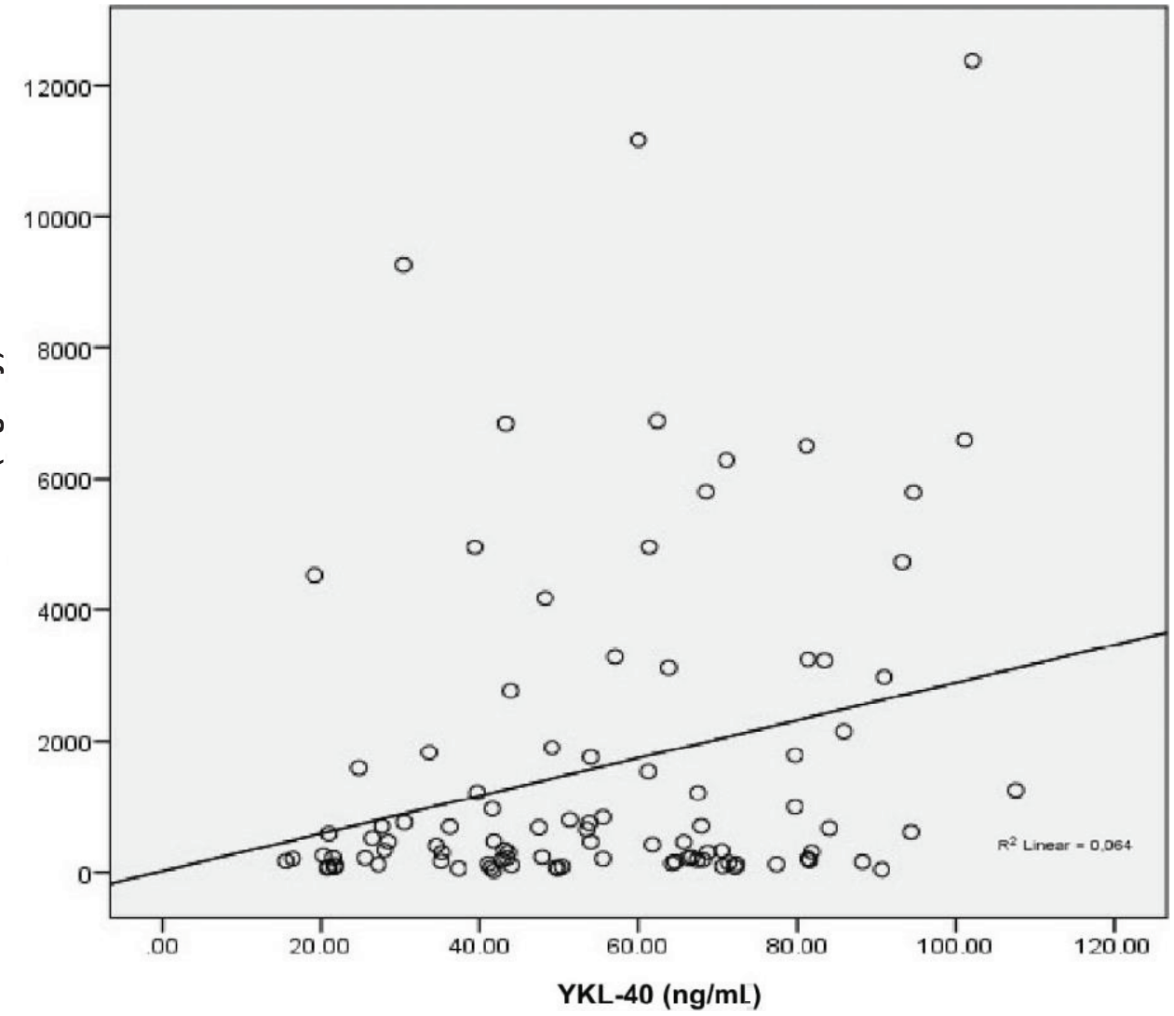
Correlation between proteinuria and YKL-40 (P < 0.013, r: 0.254).

YKL-40 and FMD did not correlate with BMI. YKL-40 had a negative correlation with FMD in multivariate analysis. Furthermore, YKL-40 was negatively associated with hemoglobin, albumin, and HDL and positively correlated with uric acid and CRP. FMD was in positive correlation with hemoglobin, albumin, and HDL while it negatively correlated with creatinine and uric acid. The analysis is summarized in Table 4. Subgroup analysis after HD patients were excluded revealed a positive association between creatinine and YKL-40 and a negative association between creatinine and FMD. As expected, the associations were vice versa for eGFR (MDRD). Box plots of FMD vs. YKL-40 and eGFR vs. YKL-40 are given in Figures 2 and 3. YKL-40 had a weak but positive correlation with proteinuria when nondialytic CKD patients were analyzed (Figure 4).

**Table 4:** Correlation analyses.

Variables	YKL-40		FMD	
	r	P	r	P
Age	0.174	0.024	–0.045	0.575
Body mass index	–0.038	0.623	–0.023	0.771
Hemoglobin (g/dL)	–0.478
<0.001	0.291	<0.001
Albumin (g/dL)	–0.429	<0.001	0.266	<0.001
Creatinine (mg/dL)	0.610	<0.001	–0.592	<0.001
eGFR (MDRD)	–0.674	<0.001	0.597	<0.001
HDL (mg/dL)	–0.366	<0.001	0.180	0.027
CRP (mg/L)	0.358	<0.001	–0.170	0.038
Glucose (mg/dL)	0.032	0.669	–0.249	0.661
Uric acid (mg/dL)	0.220	0.004	–0.237	0.002
YKL-40 (ng/mL)	-	-	–0.471	<0.001
FMD (%)	–0.471	<0.001	-	-

BP: Blood pressure, HDL: High-density lipoprotein, CRP: C-reactive protein, FMD: flow-mediated dilatation.

## 4. Discussion

In the present study, we explored the potential role of YKL-40 measurement in chronic kidney disease and dialysis patients. Our study indicates that YKL-40 levels were increased in patients with chronic kidney disease, especially hemodialysis patients. Elevated YKL-40 associated positively with CRP, creatinine, and uric acid and negatively with FMD, albumin, and hemoglobin. Importantly, our findings revealed that levels of YKL-40 correlated with eGFR. This finding also suggests that a potential mechanism for increased YKL-40 levels may be associated with reduced kidney functions.

 Cardiovascular diseases are the most common reason for death in hemodialysis patients [1]. In this study, we aimed to investigate the relationship between YKL-40 and FMD, an indirect test of endothelial dysfunction. We revealed high levels of circulating YKL-40 in both nondialytic CKD and HD patients. Furthermore, we have shown that as eGFR decreases YKL-40 increases. Our results are in concordance with the literature. Studies on CKD, HD, peritoneal dialysis, and renal transplant patients have all shown a negative association between YKL-40 and eGFR [13–15]. 

Primary cells secreting YKL-40 are macrophages and essential for acute and chronic inflammation. Thus, YKL-40 is readily accepted as an acute phase protein [16–18]. Our study reveals YKL-40’s positive association with uric acid and CRP and negative association with albumin. This association may reflect the role of inflammation in the process of endothelial dysfunction in CKD and HD patients. 

Measuring FMD by ultrasonography is a noninvasive, safe, and reproducible method to detect endothelial dysfunction and consequently to investigate preclinical atherosclerosis. Individuals, and especially patients with CKD, who have impaired endothelial function have a higher risk of CVD. It has been suggested that endothelial dysfunction facilitates the development of CVD in uremic patients. FMD decreases with age and has been shown to decrease in cases of CKD [17]. In our study patients, FMD measurements have been found lower in CKD and lowest in HD patients when compared to healthy subjects. A strong positive correlation with eGFR has also been observed. This observation makes us believe that endothelial dysfunction and inflammation go hand in hand for CKD and HD patients. Another support for our belief is uric acid and its correlations. Uric acid is a known oxidant and depletes endothelial nitric oxide [19]. Its inflammatory properties are well appreciated [20]. Kanbay et al. demonstrated that uric acid negatively correlates with FMD in a cohort of 263 nondiabetic, newly diagnosed nondialytic CKD patients [21].

Lee et al. showed that YKL-40 increases in early diabetic nephropathy and it is an independent risk factor for albuminuria. They found that fractionated excretion of YKL-40 is low in both healthy controls and diabetic patients who have estimated glomerular filtration of more than ≥60 ml/min/1.73 m2. They were also able to prove that urinary YKL-40 levels were elevated only in patients with macroalbuminuria [22]. It is unknown whether the high levels of urinary YKL-40 in macroalbuminuric patients is from reduced tubular reabsorption of YKL-40 or increased renal production of YKL-40 due to localized inflammation. Our results demonstrate that even when HD patients are not taken into consideration YKL-40 levels in patients with nondialytic CKD increase with declining eGFR. Subgroup analysis of nondialytic CKD patients proved a significant but weak correlation of YKL-40 with proteinuria. YKL-40 is a low molecular weight protein that is entirely filtered in the glomerular filtrate and then reabsorbed and catabolized in the proximal tubules [23]. We have no knowledge about the glomerular filtration and reabsorption rates of YKL-40 in nondialytic chronic kidney disease. In these situations, not only are inflammation and endothelial dysfunction prominent, but the urinary excretion and catabolism rate of YKL-40 may also be decreased. We may postulate that decreased tubular reabsorption or increased local/systemic production via inflammatory pathways or both are responsible for elevated YKL-40 levels in patients with significantly reduced eGFR. We believe both mechanisms are involved since numerous studies have shown that YKL-40 is related to metabolic syndrome and its components (hypertension, hyperlipidemia, cardiovascular and cerebrovascular diseases), various cancers, obstructive sleep apnea syndrome, chronic allograft nephropathy, and Alzheimer disease [15,24–27]. In these studies, patients with normal kidney function had elevated YKL-40 levels. That also proves that there is a threshold for tubular reabsorption and it can be exceeded with overproduction. 

Our study has several strengths. It compares patients with different degrees of renal dysfunction with healthy controls and proves that as renal dysfunction progresses YKL-40 and endothelial dysfunction also increase. There is limited English literature about YKL-40 and hemodialysis. Our study is the first to establish the relation between YKL-40 and endothelial dysfunction in HD patients. However, there are also limitations that should be mentioned. First, this is a sectional study. Therefore, no assumptions can be made about future clinical implications of endothelial dysfunction. Second, the number of HD patients is low compared to control and nondialytic CKD patients. That may have interfered with the statistics. Third, YKL-40 was only measured once, and serial measurements and their means could provide more reliable data for certain individuals. Last, due to the nature of FMD measurements, some patients with poor performance status did not volunteer for the study, which may have created a selection bias. That bias is particularly important since patients who did not wish to participate tended to have multiple comorbidities and their YKL-40 levels could have been higher than in our HD patient cohort. Had they been included, more striking differences between HD patients and other groups could have been observed.

In conclusion, YKL-40 is a new inflammatory marker that is elevated in patients with various stages of kidney disease. This study shows that YKL-40 increases with CKD stage; is negatively correlated with FMD measurements, hemoglobin, HDL, and albumin and is positively correlated with CRP and uric acid levels. We believe that YKL-40 is a significant marker to asses both endothelial dysfunction and inflammatory processes in CKD. We think that after sufficient data are obtained anti-YKL-40 antibodies may offer us a new therapeutic option against CKD and the aforementioned diseases as well.

## Acknowledgments

This study was funded by the Turkish Hypertension and Kidney Diseases Association. This study was presented as a poster at the 53rd ERA-EDTA 2016 Congress under the name “SP370-Relationship between plasma YKL-40 levels and Endothelial dysfunction”.
